# Cancer prevention trial of a synergistic mixture of green tea concentrate plus *Capsicum* (CAPSOL-T) in a random population of subjects ages 40-84

**DOI:** 10.1186/1559-0275-11-2

**Published:** 2014-01-06

**Authors:** Claudia Hanau, D James Morré, Dorothy M Morré

**Affiliations:** 1 HANAU Health Services, Inc, 160 Sagamore Parkway W, West Lafayette, IN 47906, USA; 2 MorNuCo, Inc, 1201B Cumberland Avenue, West Lafayette, IN 47906, USA

**Keywords:** Cancer, Curative prevention®, Prevention trial, Early detection, Early intervention, Green tea concentrate, Capsol-T®, ONCOblot Tissue of Origin Cancer Test

## Abstract

**Background:**

Experts agree that one of the more promising strategies in cancer management is early detection coupled with early intervention. In this study, we evaluated an early cancer detection strategy of cancer presence based on serum levels of the cancer-specific transcript variants of ENOX2 in serum coupled with an ENOX2-targeted nutraceutical preparation of green tea concentrate plus *Capsicum* (Capsol-T®) as a strategy of Curative Prevention® involving early detection coupled with early intervention in early stage cancer when in its most susceptible and manageable stages.

**Experimental design:**

One hundred ten (110) subjects were tested for cancer presence using the ONCOblot® Tissue of Origin 2-D gel/western blot protocol for detection of serum presence of transcript variants of the ENOX2 protein. Subjects testing positive for ENOX2 received 350 mg of Capsol-T® in capsule form every 4 h including during the night for periods of at least 3 to 6 months or longer after which they were again tested for ENOX2 presence using the ONCOblot® Tissue of Origin Cancer Test protocol.

**Results:**

Of the 110 subjects, both male and female, ages 40 to 84, with no evidence of clinical symptoms of cancer, 40% were positive for ENOX2 presence in the ONCOblot® Tissue of Origin Cancer Test. After completion of 3 to 17 months of Capsol-T® use, 94% of subjects subsequently tested negative for ENOX2 presence.

**Conclusions:**

Oral Capsol-T® is well tolerated and, for ENOX2 presence in serum in the absence of clinical cancer symptoms, is consistently effective in reducing the serum ENOX2 levels to below detectable limits.

## Background

Cancer is the second leading disease cause of death in the United States [1]. A group of more than l00 different and distinctive diseases, cancer may involve any tissue of the body. Estimates are that there were over l.6 million cases in 2012 in the United States alone [2]. While variable among different cancers, only a small fraction, perhaps 25% or less, are currently diagnosed at a localized stage where curative therapy is effective [3]. Most cancers are diagnosed only after the primary tumor has already metastasized so that chemotherapy is required for treatment [4]. Hence, early detection is a favored opportunity to reduce cancer mortality. By detecting cancer in its very earliest stages, when perhaps only a small number of cells are present, it is possible that early intervention will be effective in preventing further development of the incipient cancer thereby resulting in what might be viewed as Curative Prevention® [5].

Thus, despite advances in early detection of major forms of human cancer (prostate, breast, lung, colon, leukemia, lymphoma), more often than not, cancers have developed to a sufficiently late stage at the time of detection to preclude most opportunities for curative therapy [1,3]. The problem is exacerbated for pancreatic cancer, as an example, where clinical symptoms invariably are delayed until the disease state is well advanced beyond metastatic spread [1,3]. A need for early detection remains as one of the most important challenges at the forefront of cancer research, treatment and prevention.

Ecto-Nicotinamide Adenine Dinucleotide Oxidase Disulfide-Thiol Exchanger 2 (ENOX2) (GenBank accession no. AF207881) [6], also known as Tumor-Associated Nicotinamide Adenine Dinucleotide Oxidase (tNOX), is ideally suited as a target for early diagnosis of cancer as well as for early preventive intervention [5,7]. The proteins are expressed on the cell surface of malignancies and detectable in the serum of patients with cancer [8]. ENOX2 proteins are terminal hydroquinone oxidases of plasma membrane electron transport. From the standpoint of early intervention, they are important in the growth and enlargement of tumor cells [5,9-11]. Our approach, using ENOX2 as a target for both early detection and for early intervention, is based on these properties [5,7,9,12,13]. While ENOX2 presence provides a non-invasive approach to cancer detection, without methodology to identify cancer site-specific ENOX2 forms, it offered no indication as to cancer type or location.

The opportunity to simultaneously determine both cancer presence and cancer site emerged as a result of 2-dimensional gel electrophoretic separations where western blots with a pan ENOX2 recombinant single chain variable region (ScFv) antibody carrying an S tag [14] or linked to alkaline phosphatase was employed for detection. The antibody cross-reacted with all known ENOX2 forms from hematological and solid tumors of human origin but, of itself, did not differentiate among different kinds of cancers. Analyses using this antibody, when combined with two-dimensional gel electrophoretic separation, revealed specific ENOX2 species subsequently identified as transcript variants, each with a characteristic molecular weight and isoelectric point indicative of a particular form of cancer [14] (Table 1).

**Table 1:** Molecular weight and isoelectric point ranges (99 percentile) from ONCOblot data base of ENOX2 transcript variants for tissues of origin encountered in study

**Cancer**	**n**	**Molecular weight**	**Isoelectric point, pH**
Bladder	9	63-66 and 42-48 kDa	4.2-5.8 and 4.1-4.8
Blood cell	80	38-48 kDa	3.6-4.5
Breast	355	64-69 kDa	4.2-4.9
Cervical	18	90-100 kDa	4.2-5.4
Colorectal*	88	80-96, 50-60 and 33-46 kDa	4.5-5.3, 4.2-5.1 and 3.8-5.2
Melanoma	27	37-41 kDa	4.6-5.3
Mesothelioma	10	59-62 and 38-41 kDa	3.8-4.1 and 4.4-4.6
Non-small cell lung	75	53-56 kDa	4.7-5.3
Ovarian	78	72-90 and 37-47 kDa	3.7-5.0 and 3.7-5.0
Papillary thyroid	5	56-66 and 37-44 kDa	4.5-4.9 and 3.2-3.7
Prostate	79	71-88 kDa	5.1-6.5
Squamous cell	10	54-68 kDa	5.0-5.4
Uterine	9	64-69 and 36-48 kDa	4.2-4.9 and 4.5-5.6

*All three transcript variants or, frequently, only one or the other of the two higher molecular weight species, may be present.

ENOX transcript variants of specific molecular weights and isoelectric points are produced uniquely by patients with cancer [14]. The proteins are shed into the circulation and have the potential to serve as definitive, non-invasive and sensitive serum markers for early detection of both primary and recurrent cancers in at risk populations with a low incidence of both false positives and false negatives, as they are molecular signature molecules produced specifically by cancer cells and are absent from non-cancer cells. The predictive correlation between ONCOblot findings and actual onset of cancer is based on observations that a positive ONCOblot indicates the presence of ENOX2 which is a definitive marker of cancer presence.

Phase I studies with green tea concentrate have shown that green tea products may be administered safely at oral doses of up to 5 g/day [15]. Dose limiting toxicities are primarily associated with the caffeine content [15,16]. Therefore, the present study was to determine whether an oral decaffeinated green tea product might be effective in early cancer intervention with efficacy based on elimination or reduction to below the limits of detection of the cancer-specific ENOX2 proteins from subjects’ sera.

## Patients and methods

### Eligibility

A total of 110 subjects were enrolled. Subjects were recruited primarily from the Lafayette, Indiana area. Eligible subjects were males and post-menopausal females or females following an accepted method of contraception between the ages of 40 and 84 years, having no clinical evidence or history of cancer, and a body mass index between 18-35 kg/m^2^. All subjects gave their written informed consent and agreed to refrain from taking commercially available green tea supplements during the trial.

Subjects were excluded from participation if any of the following applied:

• Sexually active pre-menopausal female not using contraception

• Current diagnosis of active cancer other than superficial basal cell carcinoma (any stage)

• Less than 5 years post cancer with no evidence of disease

• Known HIV infection

• Subject had a bleeding or clotting disorder

• Participation in another clinical study involving the use of an investigational drug or product within the past 20 days

• Known allergies to supplements

No further data was collected if the following occurred

• The investigator decided to discontinue the study in the best medical interest of the subject (e.g., due to noncompliance, an adverse event, or a change in medical status)

• The subject requested withdrawal from the study

• The subject died

• The subject never received product

• The subject never met study entry criteria

• If a subject’s participation in the study was terminated prematurely, the primary reason for withdrawal was recorded and the end of study assessments were obtained, if possible.

### Product characteristics

The source of food grade green tea concentrate was a synergistic mixture of decaffeinated green tea plus *Capsicum* powder marketed under the trade name of Capsol-T® and provided as clear, oval-shaped 350 mg gel capsules. The capsules contained food grade decaffeinated green tea powder and food grade red pepper powder (*Capsicum*) in a ratio of 25:1 (green tea:C*apsicum*). The caffeine content of the 350 mg Capsol-T® product was 0.472% (1.65 mg caffeine), as determined by high performance liquid chromatography. The daily amount of caffeine for each subject was approximately 10 mg. The average cup of coffee contains between 90 and 150 mg of caffeine. A daily dose of Capsol-T® distributed over a 24 h period contained less than 10% of the caffeine of a single cup of coffee.

The vitamin K contained in natural green tea can reduce effectiveness of blood thinning medications, such as warfarin. The decaffeination process for Capsol-T® removes the vitamin K naturally present in green tea, to below levels found, for example, in lettuce.

The *Capsicum* powder component of Capsol-T® is from a relatively mild pepper (guajillo, a red pepper similar to bell peppers). This variety of pepper was specifically chosen to avoid the potential side effects and risks associated with capsaicin, a chemical ingredient found in “hot” peppers. The capsaicin content in the Capsol-T® pepper component is low or undetectable either by taste (measured as 0 scoville units; scoville units indicate the amount of capsaicin, or spicy heat) or by high performance liquid chromatographic methods, using the concentrated starting material blended with the green tea.

The Capsol-T® was prepared to meet Good Manufacturing Practice (GMP) requirements and was provided by Stratum Nutrition, a division of Novus International, St. Charles, Missouri (TeaFense).

### Study design

The study design was that of a non-randomized, open-label and IRB-approved Phase II trial of serum ENOX2 early detection followed by Capsol-T® intervention to reduce ENOX2 levels to below levels of detection in the ONCOblot® Tissue of Origin Cancer Test (ONCOblot®) protocol used for early detection. Here, we report on the first 110 subjects who satisfied all inclusion and exclusion criteria.

All study subjects were evaluated for serum presence by the standard ONCOblot® test [14]. Study subjects with serum presence of ENOX2 transcription variants were provided with Capsol-T® and instructed to consume one 350 mg Capsol-T® capsule 6 times daily, once every 3 to 5 h even during the night with a liquid of their choice other than green tea and with or without food before or after supplement consumption. They were instructed that if they missed a dose of the product, they should take the dose as soon as possible thereafter. Missed doses were recorded in the subject’s diary.

Subjects consumed the capsules daily, as instructed, for up to a 6 month period or longer, beginning at the baseline visit and continuing until their last visit and ONCOblot® retest.

### Evaluation during study

At the screening visit, demographic data were collected which included the following: age, gender, height, body weight, and calculated BMI (kg/m^2^).

During the screening period, information regarding the subject’s medical history including but not limited to, prior medication and contraception use, alcohol consumption, and smoking history to assess those characteristics that may affect efficacy/safety or study variables, was recorded.

Treatment efficacy was based on ablation of ENOX2 protein in sera of the treatment group with efficacy evaluated at 6 to 9 months compared to baseline with an optional ONCOblot® test at 3 to 5 months. Retesting was delayed for more than a year in 5 patients and 12 were not retested.

Serum samples were analyzed for the presence of ENOX2 transcript variants using the ONCOblot® test which uses 2-D gel/western blot analysis. Based on previous work, the distinct patterns of ENOX2 isoforms with characteristic molecular weights and isoelectric points (pH) from patients within this study are summarized in Table 1.

Subject compliance was monitored through review of subject diaries at Visit 2 (3 months) and at Visit 3 (6 to 9 months).

Subjects agreed to refrain from taking commercially available green tea supplements during the trial. Subjects also agreed not to take any of the medications having potential interactions with components of Capsol-T®.

Subjects were instructed to consume their normal diet during the study.

### Adverse event assessment

Following protocol guidelines, any adverse events were assessed and recorded regularly including any unfavorable or unintended sign, such as hematological and other laboratory abnormalities, symptoms temporarily associated with the use of the product, whether or not considered related to the product, including (but not limited to) those events resulting from use as stipulated in the protocol that required intervention by a health care professional, including withdrawal of product, reduced product administration, or significant additional concomitant therapy.

## Results

All 110 patients were evaluated for presence of ENOX2 transcript variants (Table 1). Their median age was 60 years (range 40-84 years) (Table 2). Forty-three patients were men. None of the patients were previously diagnosed with cancer.

**Table 2:** Patient characteristics

**Characteristics**	**Number**
Total number of patients	110
Number of patients evaluated for toxicity	32
Number of patients evaluated for response to therapy	32
Median (range) age, years	60 (40–84)
Gender: male number	43
Median (range) time from diagnosis to negative ONCOblot® retest (months)	8 (3–17)

Of the 110 subjects, 66 were negative in the ONCOblot® Tissue of Origin Cancer Test, indicative of the absence of ENOX2 transcript variants or a level of ENOX2 transcript variants below the level of detection in the test (Table 3). Of the 44 subjects with positive ONCOblot® Tissue of Origin Cancer Tests, i.e., with evidence for an ENOX2 transcript variant, non-small cell lung (20%), breast (16%), colorectal (9%), blood cell, ovarian, prostate and cervical all at 7% each were among the most prevalent based on molecular weight and isoelectric point characteristics compared to data base values from patients with clinically diagnosed cancers (Table 1). Also represented were malignant mesothelioma, squamous cell, cervical, uterine and papillary thyroid cancers.

**Table 3:** Baseline early cancer presence and result

		**ENOX2 present**		**Transcript variant**	**Retest**	**Post intervention**	
		**MW**	**pH**		**months**	**MW**	**pH**
1.	57 F	55	4.2	Colorectal	14	Negative	
		38	4.0				
2.	64 F	Negative					
3.	45 F	Negative					
4.	51 F	Negative					
5.	71 M	58	4.0	Mesothelioma (Mesothelium)	9	Negative	
		40	3.9				
6.	58 M	54	5.1	Non-small cell lung	7	Negative	
7.	59 F	75	4.8	Ovarian	8	Negative	
		40	4.6				
8.	61 F	Negative					
9.	81 F	Negative					
10.	55 F	Negative					
11.	68 F	64	4.2	Breast	7	Negative	
12.	52 M	54	5.0	Non-small cell lung	12	Negative	
13.	45 F	Negative					
14.	46 F	Negative					
15.	73 F	80	4.5	Ovarian	7	Negative	
		38	4.1				
16.	54 F	Negative					
17.	63 F	Negative					
18.	75 F	66	4.2	Breast	6	Negative	
19.	53 M	Negative					
20.	67 F	80	4.6	Colorectal	7	Negative	
		52	5.1				
21.	73 M	Negative					
22.	70 M	Negative					
23.	61 F	Negative					
24.	50 F	54	5.2	Non-small cell lung	22	54	5.2
25.	54 F	64	4.2	Breast	8	Negative	
26.	84 M	54	5.2	Non-small cell lung	5	Negative	
27.	59 F	Negative					
28.	74 M	56	4.8	Non-small cell Lung	4	Negative	
29.	64 F	58	4.5	Colorectal		No retest	
		40	4.4				
30.	61 M	Negative					
31.	52 F	Negative					
32.	83 M	Negative					
33.	75 M	Negative					
34.	51 F	43	3.8	Blood cell	17	Negative	
35.	71 F	Negative					
36.	65 M	Negative					
37.	50 F	Negative					
38.	47 M	39	4.4	Blood cell	12	Negative	
39.	54 F	50	5.1	Colorectal	12	Negative	
		38	3.8				
40.	71 M	54	5.1	Non-small cell lung		No retest	
41.	79 M	120	4.5	Not in data base	7	Negative	
		34	5.2				
42.	75 M	Negative					
43.	59 M	60	5.1	Squamous cell	7	Negative	
44.	52 F	Negative					
45.	63 F	35	4.0	Generic ENOX2	14	64	5.1
46.	60 F	Negative					
47.	58 F	Negative					
48.	61 F	68	4.4	Breast		No retest	
49.	52 F	42	3.9	Blood cell		No retest	
50.	76 M	Negative					
51.	54 F	Negative					
52.	54 F	Negative					
53.	63 F	55	5.0	Non-small cell lung		No retest	
54.	52 F	Negative					
55.	56 F	Negative					
56.	49 F	92	4.6	Cervical	3	Negative	
57.	54 F	Negative					
58.	58 M	88	5.1	Prostate		No retest	
59.	68 F	68	4.2	Breast	8	Negative	
60.	49 F	Negative					
61.	50 F	Negative					
62.	64 F	Negative					
63.	62 M	33	4.2	Generic ENOX2	9	Negative	
64.	50 F	Negative					
65.	50 M	40	5.0	Melanoma		No retest	
66.	72 F	64	4.6	Breast		No retest	
67.	49 F	Negative					
68.	50 F	Negative					
69.	54 F	54	5.0	Non-small cell lung		No retest	
70.	73 M	Negative					
71.	49 F	Negative					
72.	81 F	Negative					
73.	47 F	75	4.1	Ovarian		No retest	
		46	4.0				
74.	48 M	80	4.2	Not in data base	9	Negative	
75.	56 F	Negative					
76.	46 M	Negative					
77.	54 F	67	4.5	Uterine	3	Negative	
		39	4.9				
78.	54 F	Negative					
79.	65 M	Negative					
80.	55 M	68	4.0	Not in data base	9	Negative	
81.	60 M	83	5.7	Prostate	12	Negative	
82.	80 F	Negative					
83.	58 M	71	5.5	Prostate	12	Negative	
84.	83 M	Negative					
85.	53 M	Negative					
86.	78 F	Negative					
87.	40 F	90	5.4	Cervical	6	Negative	
88.	64 M	Negative					
89.	56 M	Negative					
90.	62 F	65	4.2	Breast		No retest	
91.	52 F	Negative					
92.	57 F	58	4.6	Papillary thyroid	7	Negative	
		44	3.7				
93.	52 F	Negative					
94.	67 M	Negative					
95.	70 M	Negative					
96.	46 M	68	5.1	Squamous cell	24	Negative	
97.	52 F	Negative					
98.	56 F	Negative					
99.	53 M	Negative					
100.	59 M	54	4.8	Non-small cell lung		No retest	
101.	66 M	Negative					
102.	70 F	Negative					
103.	59 M	Negative					
104.	77 F	92	5.1	Cervical	4	Negative	
105.	68 M	Negative					
106.	62 M	Negative					
107.	58 F	Negative					
108.	78 F	66	4.6	Bladder	3	Negative	
		44	4.6				
109.	62 M	Negative					
110.	65 M	Negative					

The 44 subjects presenting with an ONCOblot® Tissue of Origin Test with evidence for an ENOX2 transcript variant, were provided with successive 3 month supplies of Capsol-T® and retested at the interval shown. A total of 12 subjects failed to return for a retest (Table 2). Of the 32 subjects that did return for a retest, 2 (4%) remained positive for ENOX2 splice variant presence. Six patients, when retested at 3 to 5 months, were negative. An additional 16 subjects had negative ONCOblot® Tissue of Origin Tests when retested between 6 and 9 months after entering the study. For 6 subjects, where retesting was delayed until 12 to 17 months, the ONCOblot® Tissue of Origin Test was negative as was one patient that was not retested until 2 years after entering the study. With the exception of the latter patients the mean interval between admission into the study and the return of a negative ONCOblot® Tissue of Origin Test was 8 ± 4 months.

**Figure 1:**
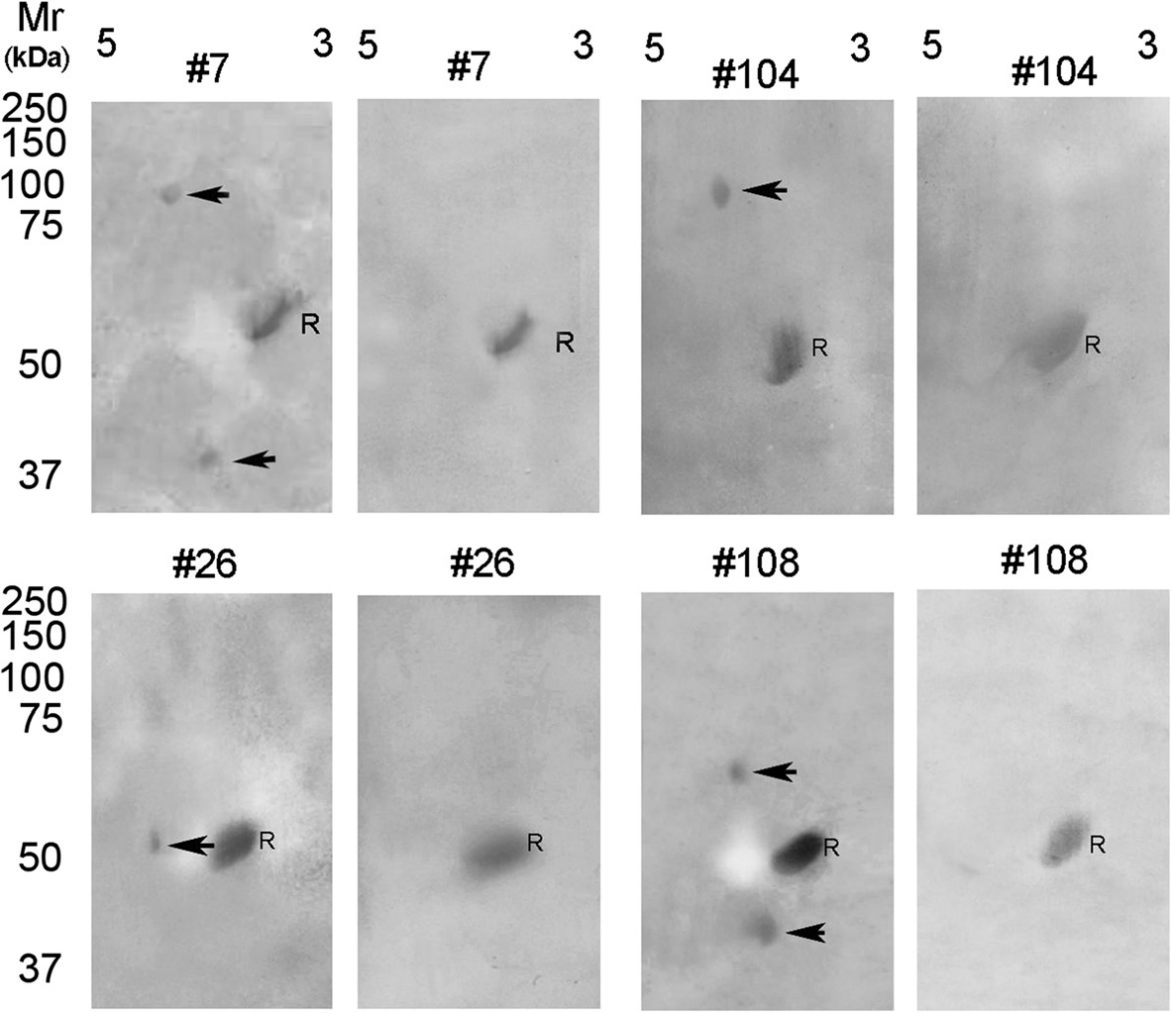
**2-D gel-western blot images of patients 7 (ovarian cancer), 26 (non-small cell lung cancer), 104 (cervical cancer), and 108 (bladder cancer).** Isoelectric focusing was in the first dimension, pH range 3 to 5 shown, with sodium dodecyl sulfate gel electrophoresis in the second dimension with comparisons to a standard reference α-fetuin (R). The initial blots are on the left for each pair where the tissue of origin-specific transcript variant proteins are indicated by arrows. Sera analyzed from the same patients post Capsol-T® intervention are shown on the right. R = α-fetuin reference protein common to all non-cancer and cancer patient sera. Molecular weights, isoelectric points and patient information are provided in Table 3.

Sera from individual patients with various forms of cancer were analyzed by 2-D gel electrophoresis and immunoblotting to assign each of the ENOX2 isoforms to a cancer of a particular tissue of origin (Table 1). Sera of breast cancer patients contained only the 64-69 kDa ENOX2, isoelectric point pH 4.2-4.9. Sera from cervical cancer patients contained a 90-100 kDa ENOX2 transcript variant, pH 4.2-5.4. Sera from patients with prostate cancer contained 71 to 88 kDa ENOX2 transcript variants of isoelectric point, pH 5.1-6.5. Sera from patients with non-small cell lung carcinoma contained a 53 to 56 kDa ENOX2 transcript variant, pH 4.7-5.3 while sera from ovarian cancer patients contained two ENOX2 transcript variants of 72 to 90 kDa and 37 to 47 kDa, both pH 3.7 to 5.0. Sera of colorectal cancer patients contained at least two of three possible ENOX2 transcript variants of 80 to 96 kDa, pH 4.5 to 5.3, or 50 to 60 kDa, pH 4.2 to 5.1 or 33 to 46 kDa, pH 3.8-5.2. A 38 to 48 kDa ENOX2 transcript variant of low isoelectric point pH 3.6 to 4.5 was characteristic of leukemias, lymphomas and other blood cell cancers. Sera of patients with malignant melanoma contained an ENOX2 transcript variant of 37 to 41 kDa, pH 4.6 to 5.3. Molecular weights and isoelectric points of other ENOX2 transcript variants associated with particular kinds of cancer encountered in the study (bladder, cervical, mesothelioma, papillary thyroid, squamous cell and uterine) are provided in Table 1. Representative paired ENOX2-positive ONCOblots with the initial blot on the left and the post-Capsol-T® ONCOblots on the right are illustrated in Figure 1.

One patient, whose ONCoblot Tissue of Origin Cancer Test showed an ENOx2 protein indicative of non-small cell lung cancer, elected not to take the Capsol-T®, withdrew from the study and was clinically diagnosed with non-small cell lung cancer 36 months later. Another patient whose original ONCOblot® Tissue of Origin Test indicated leukemia/lymphoma withdrew from the study, elected not to take the Capsol-T®, and was clinically diagnosed with lymphoma 10 months later.

Of the two patients testing positive following Capsol-T intervention, records ruled out non-compliance as an explanation. With patient 24, retrospective testing of the patient’s initial serum sample revealed that the patient’s circulating ENOX2 was resistant to Capsol-T which occurs occasionally. With patient 45, a retest after 36 months, approximately one year following formal closure of the study, returned a negative result.

Patient 45 was of interest in that the initial ONCOblot® Tissue of Origin Test result was a 35 kDa, pH 4.0 fully processed, generic ENOX2. Retesting after 14 months now revealed a 64 kDa pH 5.1 transcript variant indicative of breast cancer. Generic ENOX2 is frequently associated with early stage cancer, is not organ site specific and exhibits a uniform molecular weight of 34 ± 1 kDa (isoelectric point, pH 3.8 ± 0.1) which is always less than that of the organ site specific transcript variants. The low molecular weight is suggestive of nearly complete processing with the production of a limit, protease resistant, fragment. Such a limit fragment is common to most tissues of origin as indicated from ENOX2 fragments produced by different tissues of transgenic mice transfected with human ENOX2 cDNA [17].

Three patients, numbers 41, 74 and 80, presented with immunoreactive ENOX2 transcript variants not in the data base but tested negative 7 or 9 months later after taking the Capsol-T®.

Available data in humans from Capsol-T studies have so far indicated no clear adverse event profile for Capsol-T®. Polyphenols of green tea do block the action of the drug bortezomib (Velcade®) [18].

## Discussion

The percentage of subjects with a positive ONCOblot® Tissue of Origin Test result for ENOX2 protein presence of 40% was not unexpected in view of evidence that approximately one-half of all males and one-third of all females within the population of the United States will contract cancer between the ages of 60 and 85. Interestingly, among the ENOX2 transcript variants with the greatest incidence, 16 and 20%, were those characteristic of breast and non-small cell lung cancer, two cancers generally regarded as characterized by extended latency periods perhaps even as long as 20 years [5,19].

Unlike most published cancer markers, cancer-specific ENOX2 variants are not simply present as elevated levels of a serum constituent present in lesser amounts in the absence of cancer. The cancer-specific ENOX2 transcript variants result from cancer-specific expression of alternatively spliced mRNAs [10,11]. Neither the splice variant mRNAs nor the ENOX2 isoform proteins are present in detectable levels in non-cancer cells or in sera of subjects without cancer [5,7,14]. Multiple transcripts within a single tissue of origin may be attributed to different alternative splicing events as well or to transcription from independent promoters as shown for the glutamate transporter EAAC1 [20].

There was insufficient evidence to correlate positive ONCOblots with hereditary familial cancer or to evaluate the non-predictive power of the test. Follow up of those patients remaining in the study is ongoing.

Based on lower limits of detection on ONCOblots, we estimate detection of < 100 femtomoles of ENOX2 equivalent to 6 × 10^10^ ENOX2 molecules in 20 μl of serum requiring an estimated 2 × 10^6^ cancer cells in the body to generate, and equivalent to a 0.8 mm diameter cancer. With the size of a single cancer cell being about 10 microns, one cm^3^ of cancer would contain about 10 billion cells. While estimates vary, 7 mm diameter breast cancers were found about 50% of the time by mammography whereas tumors larger than 32 mm were required to be detected 100% of the time [21]. By comparison, a 32 mm tumor would contain approximately 1.3 trillion cells.

The most potent inhibitors of ENOX2 are EGCg [22] and the vanilloid, capsaicin [23]. The effect of EGCg on growth inhibition of HeLa cells and on inhibition of the enzymatic activity of ENOX2 was found to be synergized in the presence of other inactive green tea catechins such as epicatechin (EC). This suggested that green tea extracts might be a superior alternative to single agent EGCg preparations [24]. Additionally, a product containing a synergistic combination of decaffeinated green tea and a commercially available vanilloid-containing *Capsicum* preparation at a ratio of 25:1, resulted in a 100 fold increase in killing of cultured cancer cell lines compared to green tea alone [25].

Subsequent studies with HeLa cells indicated that one 250 mg capsule of the synergistic green tea-*Capsicum* mixture every 4 h was equivalent to drinking 16 cups of green tea every 4 h. The need for 1 capsule every 4 h is substantiated by pharmacokinetic information [26] and the knowledge that the inhibition of ENOX2 by both EGCg and by vanilloids is reversible [22]. In order to have therapeutic efficacy in selective killing of cancer cells, the catechins must be present in the culture medium at a level of about 100 nM and to inhibit ENOX2 continuously at that level for a period of 48 to 72 h [22]. If EGCg, for example, is removed and replaced by EGCg-free media, even after 8 h, the cultured cancer cells resume normal rates of growth. Similarly, normal rates of growth are resumed as EGCg is cleared from the culture medium and/or metabolized. In cell culture, the EGCg may not survive in the media for more than a few h at nanomolar concentrations. The cancer cells in vitro must be inhibited from growing for at least 48 and perhaps up to 72 h in order for apoptosis to be induced by EGCg in a majority of the cancer cells present.

Feasibility of an efficacious dosing schedule is indicated from studies with rats [26]. The results from the animal study are consistent with epidemiological studies in humans and animal experiments where benefit in prevention of cancer has been ascribed to drinking at least 10 cups of green tea per day without adverse effects [27,28]. Green tea polyphenols are absorbed after oral administration and reach their highest plasma levels after about l to 2 h after dosing both in rats [26,29,30] and in humans [31]. In the rat, the levels of EGCg reached a concentration of 12.3 nmoles/ml in plasma (12.3 μmolar) 60 min after a single oral administration of 500 mg/kg body weight of EGCg [32], which is more than l00 times the effective dose to stop the growth of tumor cells. The studies by Yang [33] show that the concentration of EGCg in the blood after 2-3 cups of green tea reached a maximum of about 0.6 μM.

In human studies of ingested catechins, 0.2% of the ingested EGCg and 0.2% to l.3% of ingested (-)-epigallocatechin (EGC) were found in plasma 90 min after ingestion [34]. Van het Hof et al. [35] determined the half-life for plasma levels of individuals drinking 8 cups of tea per day for 3 days to be 4.8 h for green tea and 6.9 h for black tea. After ingestion of green tea by human volunteers, C_max_ values were observed 1.4 to 2.4 h after ingestion with a half-life of 5 to 5.5 h [36]. These observations provided the rational basis for dosing at regular intervals of 4 h with the Capsol-T product.

Tea catechins, especially EGCg, in combination with *Capsicum* have been characterized as specific ENOX2 inhibitors inducing apoptotic cell death in cancer but not in non-cancer cell lines [22,37,38]. Safety and efficacy are well documented [39]. Safety has been the subject of a series of reports dealing with genotoxic, acute, dermal, sub-chronic short-term, teratogenic and reproductive assays e.g., [39-41]. Capsol-T® is both caffeine- and vitamin K-free and free of herbicide, pesticide and/or heavy metal residues. Its principal catechin constituent, EGCg, was affirmed to be generally recognized as safe by an independent review panel [42].

As the 2-D-western blot protocol detects cancer early, well in advance of clinical symptoms, the opportunity to combine early detection with early intervention as a potentially Curative Prevention® strategy for cancer by eliminating the disease in its very earliest stages is unique. The approach to early intervention is based on previous work in cell culture models showing that ENOX2 proteins are required to support the unregulated growth that typifies cancer cells [7]. If the growth function of ENOX2 is blocked for 48 to 72 h, the cancer cells cannot enlarge following division, cannot pass the checkpoint in G1 that monitors cell size and eventually undergo programmed cell death (apoptosis) [22,37,43]. As an early intervention strategy targeted to ENOX2, the herbal mixture of green tea and a powdered efficacious source of chili peppers (*Capsicum* species) with levels of capsaicin sufficiently low so as to not cause discomfort (Capsol-T®), emerges as being effective in reducing ENOX2 levels below the limits of detection in greater than 90% of the subjects thus far evaluated when combined with early detection based on ONCOblot® Tissue of Origin Cancer Test results.

## Competing interests

Claudia Hanau has no conflict of interest.

D. James and Dorothy Morré are co-discoverers of the cancer-specific subfamily of ECTO-NOX2 proteins upon which the ONCOblot® Tissue of Origin Test is based and which serves as the therapeutic targets for Capsol-T® intervention. They are co-owners of MorNuCo., Inc. established in 2011 to further the clinical development of both technologies. Thus far, no net financial gain has accrued from these activities.

## Authors’ contributions

CH provided oversight to subject recruitment and testing. DJM and DMM provided the ONCOblot® Tissue of Origin Assays. All authors contributed equally to data analysis and interpretation. All authors read and approved the final manuscript.
